# Macrophage migration inhibitory factor may contribute to the occurrence of multiple primary lung adenocarcinomas

**DOI:** 10.1002/ctm2.1368

**Published:** 2023-10-02

**Authors:** Wei Liu, Hao‐Shuai Yang, Fei‐Hang Zhi, Yan‐Fen Feng, Hong‐He Luo, Ying Zhu, Yi‐Yan Lei

**Affiliations:** ^1^ Department of Thoracic Surgery The First Affiliated Hospital Sun Yat‐sen University Guangzhou Guangdong China; ^2^ Department of Thoracic Surgery China‐Japan Friendship Hospital Beijing China; ^3^ State Key Laboratory of Oncology in South China, Collaborative Innovation Center for Cancer Medicine Sun Yat‐sen University Cancer Center Guangzhou Guangdong China; ^4^ Department of Pathology Sun Yat‐sen University Cancer Center Guangzhou Guangdong China; ^5^ Department of Radiology The First Affiliated Hospital Sun Yat‐sen University Guangzhou Guangdong China

## Abstract

**Background:**

This study aimed to identify the key genes involved in the development of multiple primary lung cancers.

**Methods:**

Differential expression analysis was performed, followed by comparing the infiltration levels of 22 immune cell types between multiple and single primary lung adenocarcinomas. Marker genes for epithelial cells with different proportions between the two types of lung adenocarcinomas were identified. The common genes between the marker genes and differentially expressed genes were identified. Finally, the effects of the key genes were tested on the in vitro proliferation, migration and morphology.

**Results:**

The infiltration levels of helper follicular T cells, resting NK cells, activated NK cells, M2 macrophages and resting mast cells were higher in the patients with multiple than in those with single primary lung adenocarcinomas. A total of 1553 differentially expressed genes and 4414 marker genes of epithelial cells were identified. Logistic regression analysis was performed on the 164 resulting genes. The macrophage migration inhibitory factor expression was positively associated with the occurrence of multiple primary lung adenocarcinomas. Moreover, its signalling pathway was the key pathway among the epithelial cells and multiple and single primary lung adenocarcinoma cells, and it was upregulated in lung adenocarcinoma cells. It also increased the expression of lung cancer markers, including NES and CA125, induced morphological changes in alveolar epithelial type II cells, and promoted their proliferation, migration and invasion.

**Conclusions:**

Multiple and single primary lung adenocarcinomas have different tumour immune microenvironments, and migration inhibitory factor may be a key factor in the occurrence of multiple primary lung adenocarcinomas.

## Graphical abstract

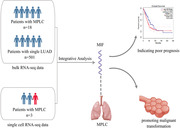

## 1 BACKGROUND

Lung cancer is one of the most common cancers worldwide, where its incidence was reported to be the second to that of breast cancer in women and prostate cancer in men in 2021. Moreover, it is the leading cause of cancer‐related death.^1^, ^2^, ^3^ Multiple primary lung cancer (MPLC) is defined as the presence of two or more primary malignant lesions in the lung of the same patient with lung cancer at the same time or successively.^4^ Based on the different time of occurrence and sequences of lesions, MPLCs can be divided into two categories: synchronous MPLCs (sMPLCs) and metachronous MPLCs (mMPLCs). The pathological diagnostic criteria for sMPLCs in the eighth edition of the TNM staging system are as follows: different lesions have (i) different histological types; (ii) significantly different semiquantitative analysis of comprehensive pathology; (iii) squamous cell carcinomas arising from carcinoma in situ; and (iv) arguments supporting sMPLCs, including different biomarker patterns and the absence of lymph node or extrapulmonary metastasis.^5^ The incidence of MPLCs has increased from 0.2% to 3.4% before 2000 and from 0.3% to 8.0% in the recent years.^6^, ^7^, ^8^, ^9^ Among the patients with operable NSCLC in China, 1%–8% are diagnosed with MPLCs after surgery.^10^, ^11^, ^12^ Surgical resection is the treatment of choice for MPLCs. Shimada et al. recommended lobectomy to remove major cancer lesions, including larger cancer lesions or invasive cancer lesions on imaging findings, and sub‐lobectomy to remove other cancer lesions. Their study revealed that there was no significant difference in the 5‐year survival between lobectomy and sub‐lobectomy for second primary lung cancer, and that sub‐lobectomy better preserved the lung function.^9^, ^13^, ^14^, ^15^ However, there is still a need for a more objective and accurate criterion to distinguish MPLCs from intrapulmonary metastatic cancers that would aid in the selection of the optimum surgical plan, as well as the determination of whether postoperative comprehensive treatment is required. Moreover, the mechanism of action of MPLCs has not yet been clarified. Studies have suggested that lung adenocarcinomas (LUAD) may originate from the lung epithelial cells.^16^, ^17^ The reasons for the development of two completely different pathways leading to multiple primary lung adenocarcinomas (MPLDs) and single primary lung adenocarcinomas (SPLDs) have not been clearly elucidated.

Macrophage migration inhibitory factor (MIF) is a cytokine that plays a key role in the immune and inflammatory responses.^18^ Studies have reported that MIF is associated with an increased risk of multiple cancers, including breast, acute myeloid, colorectal, bladder, cervical, prostate, gastric and lung cancers.^19^, ^20^, ^21^, ^22^, ^23^, ^24^, ^25^, ^26^, ^27^ Moreover, a previous study revealed that high MIF levels are associated with the risk of recurrence after lung cancer resection.^28^ Studies have also shown that MIF promotes hepatocellular carcinoma progression by regulating the immune microenvironment.^29^ MIF has also been reported to promote the growth of genitourinary malignancies, such as prostate, bladder and renal cancers, mainly through the type II transmembrane receptor CD74.^30^

Therefore, this study aimed at identifying the key implicated genes that are common between MPLDs and SPLDs using transcriptome and single‐cell analyses. It also investigated the carcinogenic effects of the key genes on normal lung epithelial cells using in vitro experiments because in vivo and in vitro models of MPLDs could not be established.

## 2 METHODS

### 2.1 Data processing

Seventeen cases including 40 LUAD lesions were collected between 2020 and 2022 at the First Affiliated Hospital of Sun Yat‐sen University. The selection criteria were as follows: (i) each cancer lesion was a primary lung cancer; (ii) the pathological report suggested that the number of lung cancer lesions was greater than or equal to two, and the lesions were adenocarcinomas; (iii) the cancer lesions were located in different lobes, the pathological subtypes of each lesion located in the same lobe were different, and no lymph node metastasis occurred, suggesting that the tumours in the same patient were of multicentric origin; and (iv) complete pathologic material was available. We downloaded the transcription data of 501 patients with SPLDs and their clinical information from The Cancer Genome Atlas (TCGA) database. Single‐cell sequencing data for MPLDs were derived from six LUAD tissues (T1, T2, T3, T4, T5, and T6) of two patients with MPLCs in the GEO database (GSE200972) and two LUAD tissues (T7 and T8) from one patient with MPLDs at our centre. Single‐cell sequencing data for SPLDs were derived from four LUAD tissues of four patients in the GEO database (three patients [P1, P2 and P3]; GSE117570 and one patient [P4]; GSE149655). This study was reviewed and approved by the Ethics Committee of the First Affiliated Hospital of Sun Yat‐sen University in strict accordance with the ethical principles of the Declaration of Helsinki. The MIF protein expression in lung cancer and normal lung tissues was obtained from the Human Protein Atlas (HPA) database.

### 2.2 Cell line and cell transfection

The lung cancer cell lines (A549, H1299, Calu‐3, PC‐9 and KTA‐7 cells) and normal human alveolar epithelial type II (AT II) cells were obtained from the American Type Culture Collection and cultured in Dulbecco's Modified Eagle medium (DMEM; Thermo Fisher Scientific). The MIF plasmids were purchased from Synechuang Bio. The AT II cells were transfected with 5 μg plasmid using lipofectamine 3000 and lipofectamine 2000 reagent (Invitrogen).

### 2.3 Bulk RNA sequencing

The total RNA was collected from fresh tissues and RNA was analysed with a Nanodrop2000 spectrophotometer (Thermo Fisher Scientific). The RNA integrity was assessed using the Agilent 2100 Bioanalyzer System (Agilent Technologies). Sample labelling and array hybridisation were performed according to the Agilent Monochrome Microarray Gene Expression Analysis Protocol (Agilent Technologies). Then, 100 μL of the hybridisation solution was dispensed into spacer slides, assembled into gene expression microarray slides, and uploaded into whole human genome expression microarrays (China National Microbiology Data Center [NMDC] ID, NMDC10018429).

### 2.4 Single‐cell RNA sequencing

Fresh tissues were processed into a single‐cell suspension using the human tumour dissociation kit and the Gentle MACS protocol. The red blood cells were subsequently removed by negative selection using CD235a beads (Miltenyi, following the recommended procedure). The number of recovered cells was determined by trypan blue exclusion using an automated counter (LUNA II). Single‐cell capture was achieved by randomly distributing the single‐cell suspensions in approximately 200 000 wells. Beads with unique molecular identifiers (UMIs) and cell barcodes were loaded close to saturation, allowing each cell to pair with the beads in the well. Single‐cell gel beads in the emulsion were created on a chromium single‐cell controller, and scRNA‐seq libraries were prepared using a chromium single‐cell 3′library and gel bead kit according to the manufacturer's protocol (10× genomics). Sequencing libraries were quantified using a high‐sensitivity DNA chip (Agilent) on Bioanalyzer 2100 and Qubit high‐sensitivity DNA assay (Thermo Fisher Scientific). Libraries were sequenced on NovaSeq6000 (Illumina) using 2× 150 chemical reagents. The BD Rhapsody analysis pipeline was used to process raw sequencing data, which were uploaded to whole‐genome expression microarrays (China National Microbiology Data Center [NMDC] ID, NMDC10018429).

### 2.5 Differential expression analysis between SPLDs and MPLDs and enrichment analysis of the differentially expressed genes

We identified the differentially expressed genes (DEGs) by comparing the LUAD tissues of 501 patients with SPLDs from the TCGA dataset and the LUAD tissues of 17 patients from our centre with a threshold for false discovery rate of less than .05, along with |log2 FC (fold‐change) greater than 2 using the R package 'edgeR'. The data were visualised using a heatmap with R package 'pheatmap'. We utilised the R packages 'clusterProfler', 'org.Hs.eg.db', 'enrichplot' and 'ggplot2' to perform Kyoto encyclopedia of genes and genomes (KEGG) and gene ontology (GO) enrichment analyses to investigate the biological processes associated with these DEGs at a significance level of *p* < .05.

### 2.6 Investigation of tumour immune microenvironment

CIBERSORT (cell‐type identification by estimating relative subsets of RNA transcripts) was used to estimate the infiltration levels of 22 immune cells in a new sample by deconvolution based on a prespecified expression profile of 22 immune cells.^31^ Using the expression profile of LUAD samples retrieved from the TCGA databases and our centre, we computed, compared and visualised the infiltration levels of 22 immune cells between the patients with SPLDs and those with MPLDs using the CIBERSORT run code supplied by the developer and the R packages 'limma' and 'ggpubr'. The ESTIMATE algorithm was used to calculate the matrix, immune and ESTIMATE scores for each sample based on one‐sample gene enrichment analysis. The stromal, immune and ESTIMATE scores reflect the content of stromal cells in the tumour, the content of the immune cells in the tumour and the purity of tumour cells, respectively.^32^

### 2.7 Single‐cell RNA‐seq quality control, dimension reduction and unsupervised clustering

The R packages 'Seurat' and 'singleR' were used to analyse four SPLD and eight MPLD samples, respectively. The cells that expressed less than 500 genes, more than 6000 genes or over 2% of the mitochondrial genes were removed. Then, the scRNA‐seq data were normalised by the 'NormalizeData' function. Based on identifying the top 2000 most variably expressed genes by the 'FindVariableFeatures' function, the principal component analysis (PCA) was performed by the 'RunPCA' function, and the principal components were summarised using uniform manifold approximation and projection (UMAP) analysis dimensionality reduction by the 'RunUMAP' function. Differential gene expression analysis was performed on each cluster to identify the marker genes for each cluster by the 'FindMarkers' function. The clusters were then classified based on reference datasets from the Human Primary Cell Atlas and CellMarker 2.0 (http://bio‐bigdata.hrbmu.edu.cn/CellMarker/) using the R package 'singleR'.^33^, ^34^

### 2.8 Selection of key markers of MPLDs

We identified marker genes of epithelial cells, including AT II, ciliated and club cells, which accounted for different proportions of SPLDs and MPLDs tissues, along with |log2 FC| > 2. The common genes between the marker genes and DEGs were retrieved. Afterwards, logistic regression analysis was performed on the resulting genes. We defined MPLDs as 1 and SPLDs as 0 for the dichotomous variables to identify the key markers of the occurrence of MPLDs rather than SPLDs.

### 2.9 Construction of cell‐to‐cell interaction networks

We used the R package 'CellChat' to investigate the cell‐to‐cell interactions between SPLDs and MPLDs. The CellChat object was created using the 'createCellChat' function. The CellChatDB.human served as a reference, and the 'identifyOverExpressedGenes' function was used to find highly expressed coreceptors in each subset. The 'identifyOverExpressedInteractions' function was used to identify the overexpressed coreceptor interactions, and the 'projectData' function was used to project the gene expression data onto the protein–protein interaction (PPI) networks. The 'ComputeCommunProb' and 'computeCommunProbPathway' functions were used to infer the aggregated communication network between the cell interaction and computational cells.

### 2.10 RNA extraction and quantitative real‐time PCR

Total RNA from tissues and cells was extracted and reverse transcribed using the TRIzol Kit (Invitrogen) and BestarTM qPCR RT Kit (DBI) by the manufacturer's protocol. The quantitative real‐time polymerase chain reaction (qRT‐PCR) was performed using a Stratagene Real‐Time PCR instrument (Agilent). The relative mRNA expression was normalised to that of the housekeeping gene, GAPDH. The primer sequences were as follows: MIF forward primer: 5′‐GTTCCTCTCCGAGCTCACC‐3′; MIF reverse primer: 5′‐TGCTGTAGGAGCGGTTCTG‐3′; GAPDH forward primer: 5′‐AACGGATTTGGTCGTATTGGG‐3′; and GAPDH reverse primer: 5′‐CCTGGAAGATGGTGATGGGAT‐3′.

### 2.11 Western blot analysis

Whole‐cell lysates were prepared and quantified following the standard protocols; then they were separated by sodium dodecyl sulfate‐polyacrylamide gel electrophoresis, and transferred onto nitrocellulose membranes. The membranes were blocked with blocking buffer and incubated with monoclonal antibodies. Afterwards, the membranes were incubated with peroxidase‐conjugated anti‐rabbit secondary antibodies. The protein bands were detected using internal control antibodies (glyceraldehyde‐3‐phosphate dehydrogenase, GAPDH). The dilutions of MIF, carcinoembryonic antigen (CEA), NES and CA125 antibodies used for Western blot were 1:1000, while the dilution of GAPDH antibody was 1:5000.

### 2.12 Enzyme‐linked immunosorbent assay (ELISA) analysis

The expression of CEA and CA125 proteins was detected using the Human CEA and Human CA125 enzyme‐linked immunosorbent assay (ELISA) kit according to the manufacturer's instructions. Approximately 50 μL of the cell suspension was loaded into the wells of a 96‐well plate, and 50 μL of the enzyme labelling reagent and 10 μL of the detection solution were added to it. After 30 min of incubation, 50 μL of the colourants A and B were added in sequence, and the optical density (OD) was measured with a microplate reader at 450 nm.

### 2.13 Haematoxylin–eosin staining

We passed the cells from different groups onto different slides, stained each slide with haematoxylin, washed them with 1% hydrochloric acid alcohol for several seconds after approximately 5 min. Then, we washed them back to blue with 0.6% ammonia followed by running water. The cytoplasm was stained with eosin for approximately 5 min on each slide. The slides were sequentially dehydrated in 95% ethanol, absolute ethanol and xylene until they became transparent. Afterwards, they were dried and mounted with neutral resin.

### 2.14 Cell proliferation detection

Cell proliferation was detected using a Cell Counting Kit‐8 (CCK‐8) assay according to the manufacturer's instructions. We loaded approximately 100 μL of the cell suspension into the wells of a 96‐well plate and added CCK‐8 solution and 10 μL of the detection solution to it. After 4 h of incubation, the OD was measured at 450 nm using a microplate reader.

### 2.15 Transwell migration and invasion assays

Transwell chambers (Corning) were used to assess the cell migration and invasion. Briefly, cells were incubated in the upper chamber with 200 μL of the medium without fetal bovine serum (FBS). The lower chamber was filled with a mixture of 80% FBS‐free medium and 20% FBS. We then incubated the cells at 37°C for 24 h, and then fixed them with 4% formaldehyde. The cells were observed under a light microscope after staining with 0.5% crystal violet.

### 2.16 Statistical analysis

Data were presented as mean ± SEM and analysed using Graphpad prism 9 software. Comparisons between groups were performed using unpaired Student's *t*‐tests for each two independent groups and one‐way analysis of variance for more than two groups. Survival analysis was performed using Kaplan–Meier survival curves. All experiments were performed three or more times. Statistical significance was set at a *p*‐value of less than .05.

## 3 RESULTS

### 3.1 Functional enrichment analysis of DEGs

**FIGURE 1:**
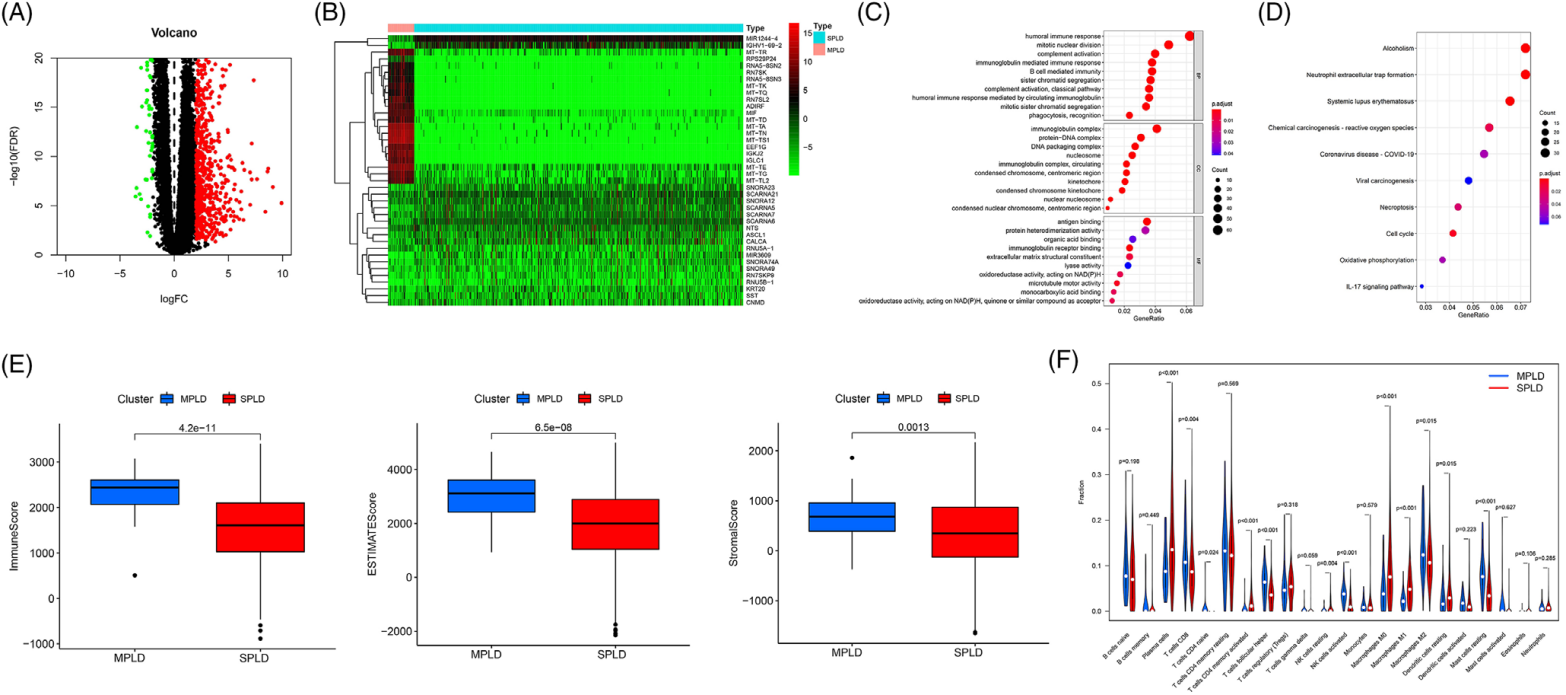
(A) Volcano map of differentially expressed genes (DEGs) between single primary lung adenocarcinomas (SPLD) and lung adenocarcinomas (LUAD) samples. (B) Heatmap of DEGs between SPLD and LUAD samples. (C and D) Bubble plots of GO analyses (C) and KEGG analyses (D). (E) The comparison of immune‐related scores including immune score, estimated score and stromal score between SPLD and LUAD. (F) The infiltrating levels of 22 immune cell types in SPLD versus multiple primary lung adenocarcinomas (MPLD).

Differential expression analysis between the MPLD and SPLD samples resulted in 1553 DEGs visualised in volcano plots (Figure 1A). Of these 1553 DEGs, 891 were upregulated in MPLDs, whereas the remaining were downregulated. The top 20 upregulated and downregulated genes are shown in a heat map (Figure 1B). GO analysis revealed that humoral immune response, immunoglobulin complex and antigen binding biological processes were significantly enriched (Figure 1C). KEGG analysis showed that alcoholism, neutrophil extracellular trap formation, systemic lupus erythematosus, cell cycle and chemical carcinogenesis pathways were significantly enriched (Figure 1D).

### 3.2 Estimation of tumour immune microenvironment

Patients with MPLDs had higher immune, estimated and stromal scores than those with SPLDs (Figure 1E). Moreover, the infiltration levels of helper follicular T cells, M2 macrophages, activated NK cells, resting NK cells and resting mast cells were higher in the patients with MPLDs than in those with SPLDs. Patients with SPLDs exhibited higher infiltration of plasma cells, CD8 T cells, activated memory CD4 T cells, naïve CD4 T cells, M0 macrophages, M1 macrophages and resting dendritic cells than those with MPLDs (Figure 1F).

### 3.3 A single‐cell atlas of the SPLDs and MPLDs

**FIGURE 2:**
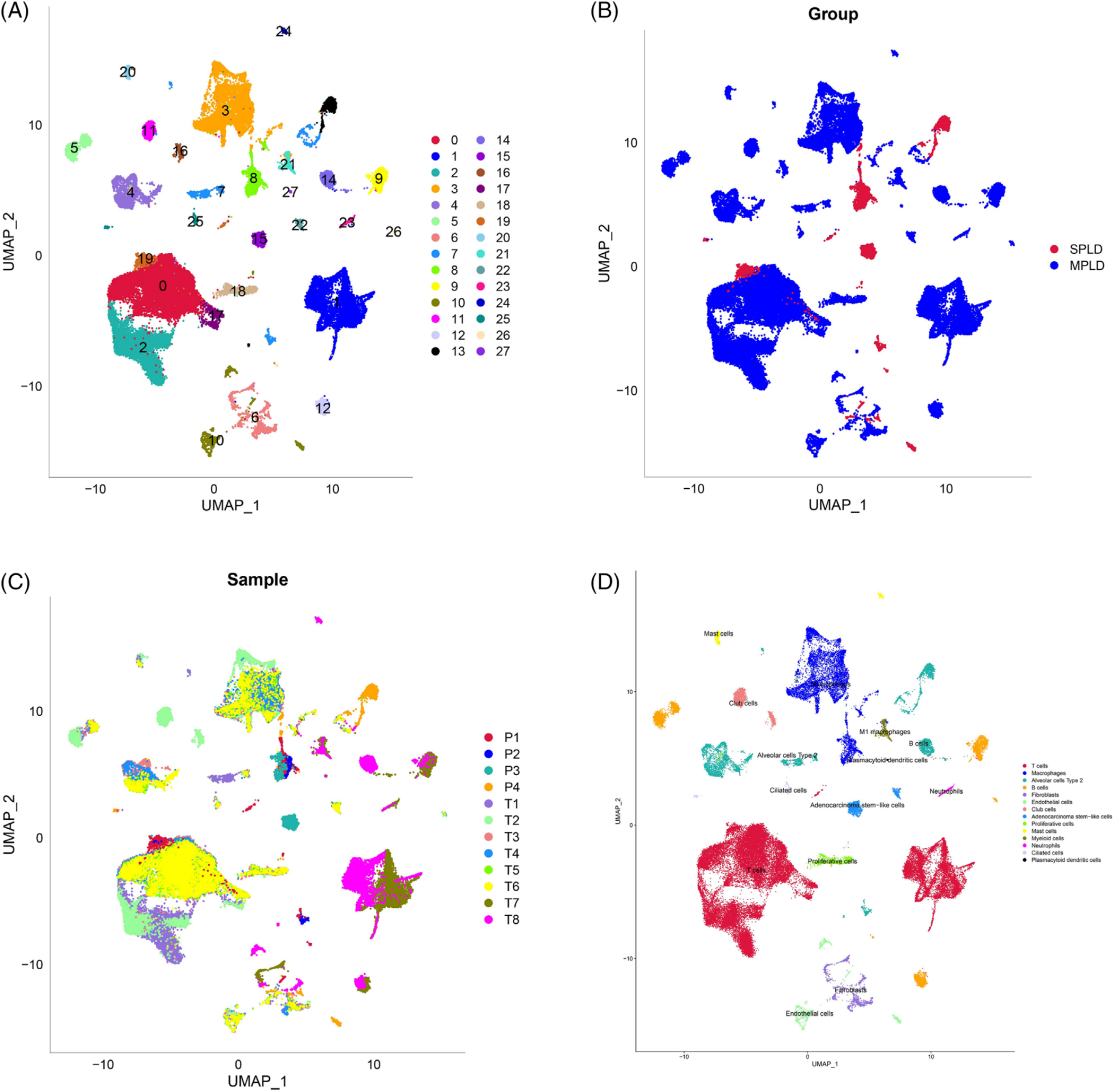
(A–D) Overview of scRNA‐seq data of four single primary lung adenocarcinomas (SPLD) and eight multiple primary lung adenocarcinomas (MPLD) samples. (A) Uniform manifold approximation and projection (UMAP) plot of 62 368 cells coloured by various cell clusters. (B) UMAP plot of SPLD samples and MPLD samples. (C) UMAP plot of each lesion sample. (D) Annotations for 14 cell types.

After quality control, the gene expression profiles of 62 368 cells, of which 57 500 and 4868 cells were derived from four SPLD and eight MPLD samples, respectively, were subjected to downstream analyses. The distributions of SPLDs and MPLDs cells are shown in the UMAP plots (Figure 2A,B). The distribution of each lesion cell is shown in the UMAP plot (Figure 2C). The clusters were further annotated using the marker genes of each cluster, and 28 clusters were assigned to 14 cell types, namely T cells, macrophages, alveolar epithelial type II (AT 2) cells, B cells, fibroblasts, endothelial cells, club cells, adenocarcinoma stem‐like cells, proliferative cells, mast cells, myeloid cells, neutrophils, ciliated cells and plasmacytoid dendritic cells (Figure 2D). T cells, B cells and fibroblasts had a higher proportion in the MPLDs tissues than in the SPLDs tissues, while macrophages, AT II cells, endothelial cells, adenocarcinoma stem‐like cells and ciliated cells had a higher proportion in the SPLDs tissues than in the MPLDs tissues (Table S1). Neutrophils, in addition to club, proliferative, mast, myeloid and plasmacytoid dendritic cells were only observed in the MPLDs tissues (Table S1).

### 3.4 Selection of key genes of MPLDs

Studies have suggested that LUAD may originate from lung epithelial cells such as AT II cells, bronchioalveolar stem cells or club cells.^16^, ^17^ Accordingly, the different directions of malignant transformation of critical epithelial cells could be responsible for the differences in the occurrence of MPLDs and SPLDs. Therefore, 4414 marker genes in the epithelial cells, including AT II, ciliated and club cells, which accounted for different proportions in the SPLDs and MPLDs tissues, were identified. Then, logistics regression analysis was performed on the 164 resulting genes, which were common between the marker genes and DEGs. The macrophage MIF expression was positively associated with the occurrence of MPLDs.

### 3.5 Cell communication analysis

**FIGURE 3:**
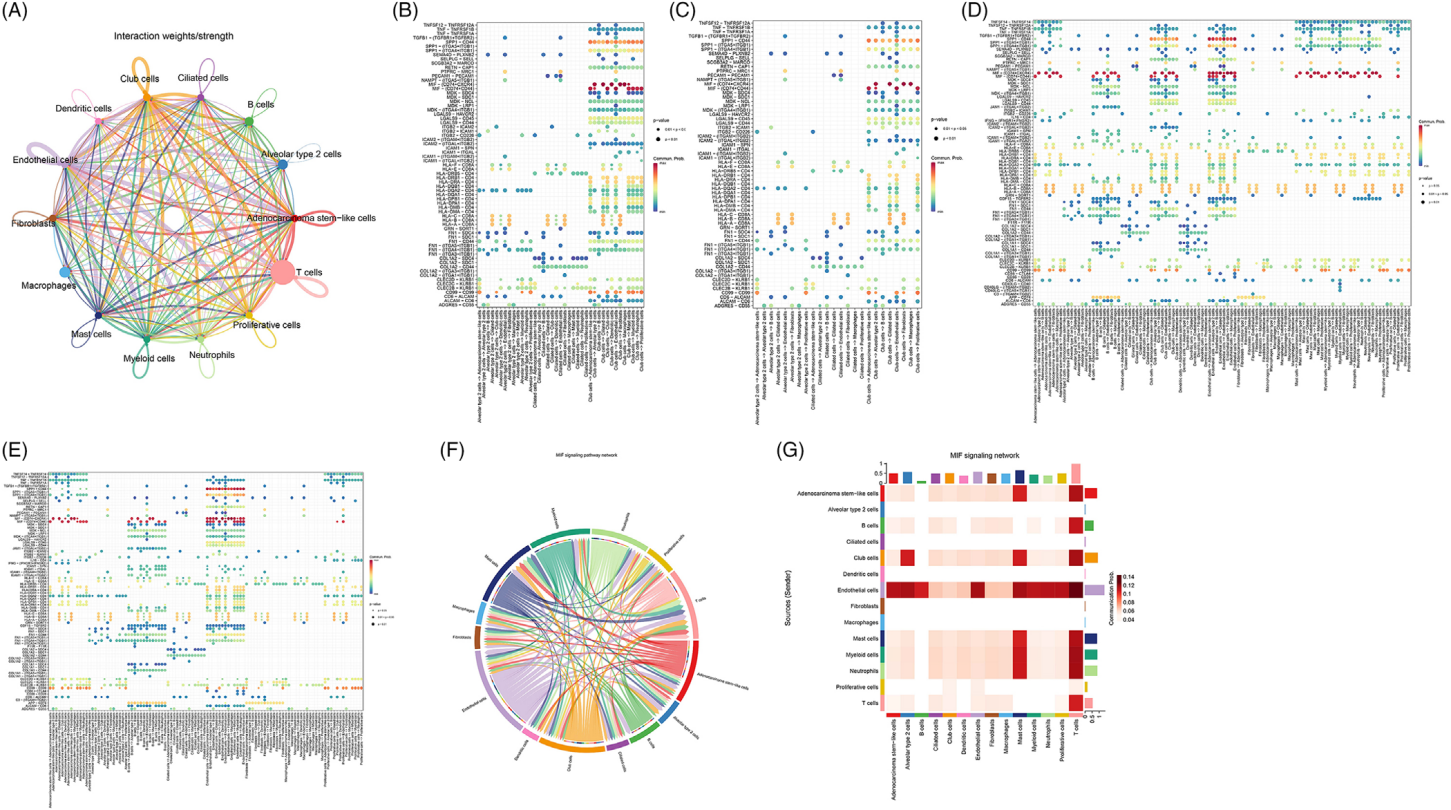
(A) Cell communication interactions between the 14 cell types. (B–E) Match‐receptor‐mediated action bubble maps between different signal‐producing cells and target cells. (B) Epithelial cells were signal‐producing cells and multiple primary lung adenocarcinomas (MPLDs) cells were target cells. (C) Epithelial cells were signal‐producing cells and single primary lung adenocarcinomas (SPLDs) cells were target cells. (D) MPLDs cells were signal‐producing cells and SPLDs were target cells. (E) SPLDs cells were signal‐producing cells and MPLDs were target cells. (F) Network of MIF signalling pathway connections among interactions across all the cell types. (G) Heatmap of the MIF signalling pathways between different cell types.

To further understand the roles and connections between the different cell types in SPLDs and MPLDs, cell communication analysis was performed. Tight interactions were observed between the 14 cell types (Figure 3A). Using epithelial cells as signal‐producing cells and MPLD cells as target cells, the resulting match‐receptor‐mediated action bubble maps showed that the most significant pathways were the MIF match‐receptor signalling pathways and that the epithelial cells were mainly club cells (Figure 3B). Using epithelial cells as signal‐producing cells and SPLD cells as target cells, similar results were observed (Figure 3C). Using MPLD cells as signal‐producing cells and SPLDs as target cells, the resulting match‐receptor‐mediated action bubble maps showed that the most significant pathways were the MIF match‐receptor signalling pathways and the SPP1 pathway (Figure 3D). Using SPLDs cells as signal‐producing cells and MPLDs as target cells, the resulting match‐receptor‐mediated action bubble maps showed that the most significant pathways were also the MIF match‐receptor signalling pathways and SPP1 pathway (Figure 3E). Figure 3F shows the network of MIF signalling pathway connections among interactions across all the cell types. A heatmap of the MIF signalling pathways between different cell types showed that endothelial, club, adenocarcinoma stem‐like, mast, myeloid, neutrophils, T and B cells transmitted the MIF signals to other cells (Figure 3G).

### 3.6 MIF was upregulated in MPLDs

**FIGURE 4:**
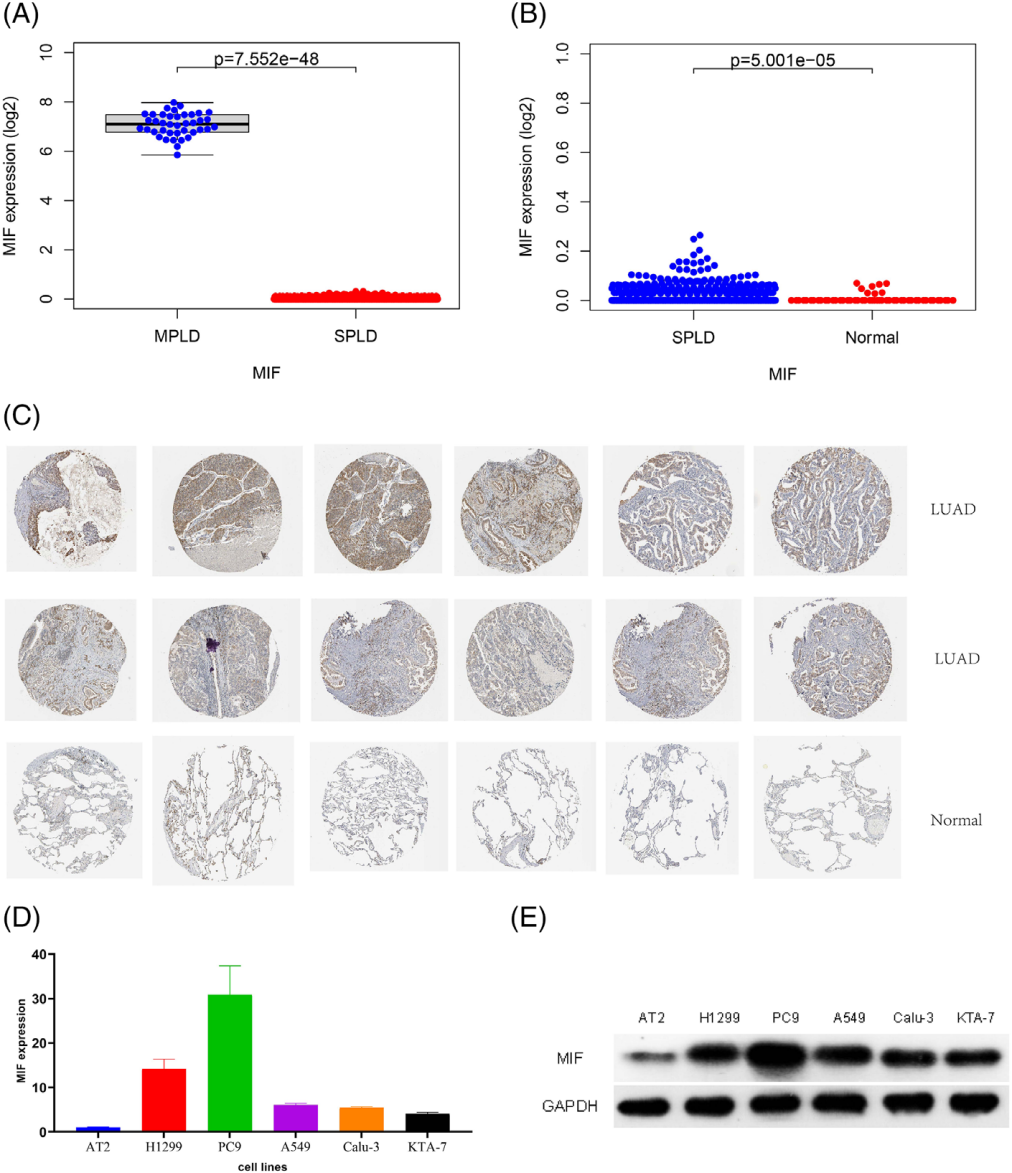
(A) Differential expression of MIF between multiple primary lung adenocarcinomas (MPLD) from our centre and single primary lung adenocarcinomas (SPLD) samples from TCGA database. (B) Differential expression of MIF between SPLD and normal samples from TCGA database. (C) Immunohistochemical analysis of MIF protein in lung adenocarcinomas (LUAD) and normal samples from HPA database. (D and E) MIF expression was compared between AT II cells and LUAD cells including A549, H1299, Calu‐3, PC‐9 and KTA‐7 cells by RT‐PCR (D) and WB (E).

The MIF expression levels were upregulated in MPLDs compared to those in SPLDs (Figure 4A). The MIF expression was further investigated in MPLDs, SPLDs and normal lung samples. SPLDs had a higher expression of MIF than the normal lung tissues (Figure 4B). Moreover, the MIF protein expression was higher in 12 LUAD tissue samples than that in six normal lung tissue samples from the HPA database (Figure 4C). We investigated the MIF expression in LUAD and normal alveolar epithelial cells. RT‐PCR analysis showed that MIF transcript expression was higher in A549, H1299, Calu‐3, PC‐9 and KTA‐7 cells than that in AT II cells (Figure 4D). Western blot analysis showed that MIF protein expression was higher in A549, H1299, Calu‐3, PC‐9 and KTA‐7 cells than in AT2 cells (Figure 4E). Both RT‐PCR and Western blot results revealed that MIF expression had the highest expression levels in PC‐9 cells. Accordingly, the differential development of MPLDs and SPLDs could be attributed to the different degrees of MIF overexpression. We further investigated whether MIF overexpression promotes the carcinogenesis of normal lung epithelial cells in vitro because the in vivo and in vitro models of MPLDs could not be established.

### 3.7 MIF promoted the increased expression of lung cancer markers and morphological changes in AT II cells

**FIGURE 5:**
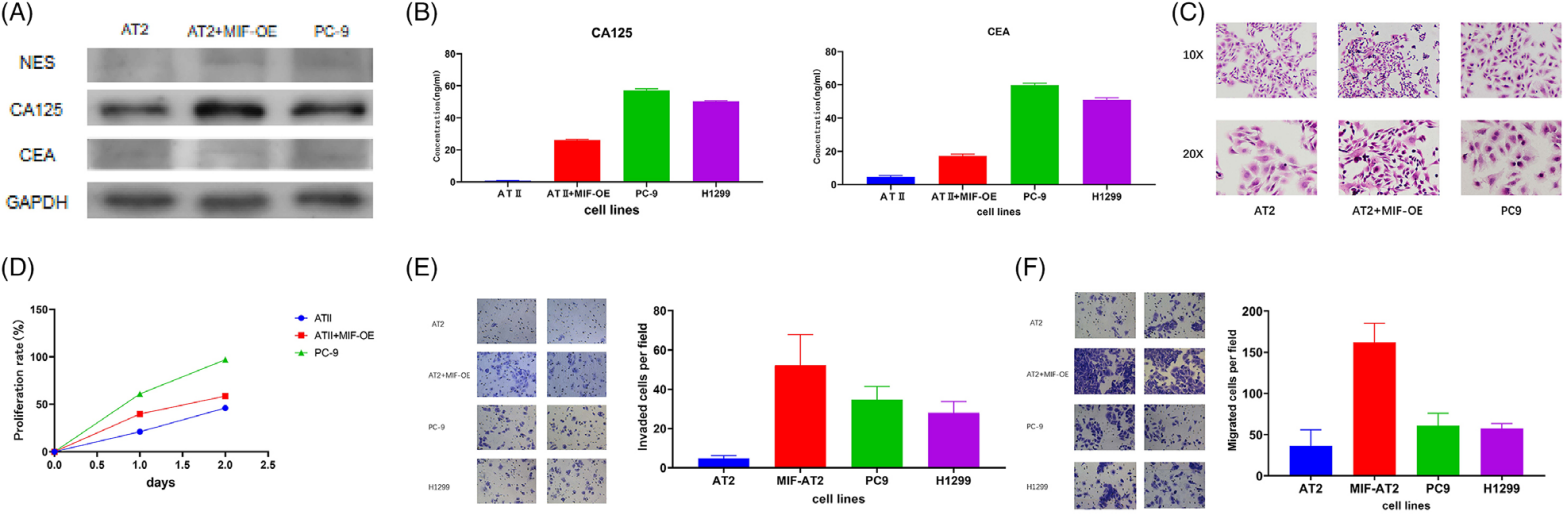
(A) WB comparing PC‐9 cells and AT II‐overexpressing cells with their respective control cells (AT II) are seen in relative expression of NES, CA125, CEA and GAPDH was taken as control. (B) ELISA comparing H1299 cells, PC‐9 cells and AT II‐overexpressing cells with their respective control cells (AT II) are seen in relative expression of CA125 and CEA. (C) Haematoxylin–eosin (HE) staining comparing PC‐9 cells and AT II‐overexpressing cells with their respective control cells (AT II) in 10× and 20×. (D) Cell proliferation rate was detected by CCK8 and statistically analysed. (E and F) MIF promoted invasion and migration of AT II cells. Representative images of migrated (E) or invasive (F) cells including H1299 cells, PC‐9 cells, AT II‐overexpressing cells and their respective control cells (AT II) are shown, and results are statistically analysed.

Western blot and ELISA results showed that the levels of CEA and CA125 were significantly increased after the overexpression of MIF in AT II cells and were lower than those in the H1299 and PC‐9 cells (Figure 5A,B). Haematoxylin–eosin (HE) staining showed that the AT II cells had clear structures, no hyperchromatic nuclei, high adhesion between the cells and a normal nucleocytoplasmic ratio. The PC‐9 cell structure was not clear, and most cells had large and hyperchromatic nuclei, an abnormal nucleocytoplasmic ratio, scattered growth and low intercellular adhesion, which are typical characteristics of malignant tumour cells. Moreover, some AT II cells overexpressing MIF had large and hyperchromatic nuclei and abnormal nucleocytoplasmic ratios (Figure 5C).

### 3.8 MIF promoted the proliferation of AT II cells

The CCK‐8 assay results revealed that MIF upregulation promoted the proliferation of AT II cells. Although the proliferation rate of AT II cells overexpressing MIF showed a significant increase, it was still lower than that of the PC‐9 cells (Figure 5D). The results of the transwell assay demonstrated that the overexpression of MIF increased the migration and invasion of AT II cells, which previously had a minimal invasive ability. The invasive ability of the AT II cells overexpressing MIF was consistent with that of LUAD cells, including PC‐9 and H1299 cells (Figure 5E). Moreover, the MIF‐overexpressing AT II cells migrated far more than the AT II and LUAD cells, including PC‐9 and H1299 cells (Figure 5F).

## 4 DISCUSSION

Lung cancer is one of the most aggressive cancers in the world.^35^ Despite the increase in the detection rate of MPLCs due to the widespread physical examination and use of computed tomography (CT), there are still disputes regarding the diagnosis, differential diagnosis and clinical management strategies of MPLCs.^8^, ^9^ However, the mechanism underlying MPLCs formation has not yet been elucidated and requires further exploration.

Our study showed significant differences in the gene expression between MPLDs and SPLDs. The enrichment results showed that the DEGs were mainly enriched in the immune‐related pathways, suggesting that differences in the developmental processes between MPLDs and SPLDs may be caused by immune‐related pathways. Therefore, we compared and analysed the tumour immune microenvironment between MPLDs and SPLDs. The M2 macrophages were more abundant in MPLDs. Previous studies have reported that M2 macrophages have immunosuppressive functions and promote tumour development.^36^ Moreover, the SPLDs had a higher number of immune effector cells, such as plasma, CD8^+^ T and activated memory CD4^+^ T cells, in addition to M0 and M1 macrophages.^37^ Therefore, MPLDs could be in a state of marked immunosuppression relative to SPLDs, which could contribute to the development of synchronous or metachronous multiple primary LUADs that form MPLDs.

Moreover, we searched for key genes that could be involved in the development of LUAD to MPLDs using single‐cell RNA‐seq. Club and AT II cells could lead to LUAD, where club cells lose the lineage fidelity following epigenetic alterations and acquire an AT II‐like phenotype following an oncogenic transformation.^38^ The development of KRAS mutations and TP53 deficiency in club cells could induce invasiveness in LUAD.^39^ Tumour‐initiating cells in LUAD are highly controversial; however, most researchers believe that LUAD is a malignant tumour arising from pulmonary glandular epithelial cells.^40^ Our study revealed that MPLDs had a higher proportion of epithelial cells, including ciliated and club cells, and a lower proportion of AT II cells than those observed in SPLDs. Afterwards, the common genes between the DEGs obtained from the transcriptome difference analysis and marker genes of these three epithelial cell types were obtained. This was followed by performing logistic regression, and a positive correlation between MIF expression and the occurrence of MPLDs was obtained. The results of cell communication showed that the MIF signalling pathway was the main interaction pathway in epithelial, MPLDs and SPLD cells, and that epithelial cells were mainly club cells. Previous studies have reported that AT II cells promote the progression of LUAD by secreting TNF‐α to upregulate the expression of MIF and CD74 in macrophages.^41^ Our results suggest that MIF and its related pathways may be important factors in the development of epithelial cells in different directions of malignant transformation, including MPLDs and SPLDs.

MIF is involved in the cell‐to‐cell immune and inflammatory response regulation by encoding lymphokines linked to inflammatory diseases and cancer.^42^, ^43^ Combined with gene expression in the normal lung tissue and LUAD tissue from the TCGA database and gene expression in the MPLDs tissues from our centre, MIF levels were found to be upregulated in SPLDs compared with those in the normal lung tissue, and the levels of MIF were further increased in MPLDs. Moreover, RT‐PCR and Western blot results revealed that MIF expression was higher in LUAD cells than that in AT II cells. Previous studies have reported that MIF is overexpressed in various tumours, such as prostate, breast, gastric and lung cancer.^44^, ^45^, ^46^, ^47^ Therefore, we speculated that the abnormal upregulation of MIF may lead to the development of LUAD, and significant upregulation of MIF may lead to the development of MPLDs. Similarly, high MIF expression has been reported to be related to poor prognosis and a high risk of recurrence in lung cancer.^28^, ^48^ We then investigated whether MIF promotes the occurrence of LUAD in an in vitro model and whether the upregulation of MIF leads to carcinogenesis in AT II cells. Our results showed that MIF promoted the increased expression of lung cancer markers, including NES and CA125, in AT II cells and carcinoid morphological changes in AT II cells, and that MIF promoted the proliferation, invasion and migration of AT II cells. Previous studies have reported that MIF promotes tumour cell proliferation and migration. MIF stimulates the proliferation of hepatocellular carcinoma cells, such as Hepa 1–6 and HepG2 cells.^49^ The long non‐coding RNA MIF‐AS1 promotes breast cancer cell proliferation, migration and epithelial–mesenchymal transition (EMT) process.^50^ MIF has also been reported to promote the invasion and growth of pancreatic cancer cells through the targeted regulation of NR3C2.^51^ Moreover, it induces the rapid phosphorylation of ERK1/2 and Akt, increases the expression of cyclin D1 and cyclin E in AT II cells, and promotes the proliferation of AT II cells.^52^ Combined with our previous findings, these results suggest that MIF may act as a carcinogenic initiator in LUAD.

Although we used multiple approaches to claim that MIF may be a key gene in the differential formation of MPLDs and SPLDs, this study has several limitations. First, the follow‐up time of the patients with MPLDs at our centre was not sufficient. Therefore, we could not investigate the association between MIF and the prognosis of MPLDs. Second, the sample size of the patients with MPLDs was relatively small. Moreover, currently there is no accepted animal model for MPLDs, and we cannot verify whether the overexpression of MIF in lung epithelial cells leads to MPLD development in vivo.

## 5 CONCLUSION

In summary, MPLDs and SPLDs had different infiltration levels of immune cells, and MIF may be a key factor in the occurrence of MPLDs. Our study improves the understanding of the development of MPLDs and SPLDs and provides new insights into the role of MIF in the development of MPLDs. Further studies, including in vivo verification, are necessary to validate our findings, which will be beneficial for the diagnosis and treatment of MPLCs.

## CONFLICT OF INTEREST STATEMENT

All authors have completed the ICMJE uniform disclosure form. The authors declare that there are no conflicts of interest regarding the publication of this article.

## Supporting information

Supporting Information

## Data Availability

The original data presented in the study are included in the article, which are available from the corresponding author upon reasonable request.
