# Supplementary material for: Macrophage migration inhibitory factor may contribute to the occurrence of multiple primary lung adenocarcinomas
Source: Clin Transl Med. 2023 Oct 2;13(10):e1368. doi: 10.1002/ctm2.1368 (PMC10545892; doi:10.1002/ctm2.1368)
Supplement: Supplementary file 1 — Supporting Information [file CTM2-13-e1368-s001.docx]

| Cell type | Number of this cell type in MPLD | Number of this cell type in LUAD | P value | Fold-change |
| --- | --- | --- | --- | --- |
| T cells | 34084 | 759 | 0.00E+00 | +7.8801088 |
| Macrophages | 6604 | 1377 | 5.02E-200 | -0.3289571 |
| Alveolar Type 2 cells | 4998 | 1317 | 3.59E-272 | -0.256676 |
| B cells | 4218 | 76 | 2.92E-72 | +4.9914793 |
| Fibroblasts | 1458 | 92 | 4.63E-03 | +1.3505802 |
| Endothelial cells | 834 | 275 | 3.45E-68 | -0.2458144 |
| Club cells | 1947 | 0 | 3.09E-70 | Inf |
| Adenocarcinoma stem-like cells | 461 | 895 | 0.00E+00 | -0.0358777 |
| Proliferative cells | 808 | 0 | 4.23E-29 | Inf |
| Mast cells | 849 | 0 | 1.04E-30 | Inf |
| M1 macrophages | 493 | 0 | 6.32E-18 | Inf |
| Neutrophils | 452 | 0 | 1.53E-16 | Inf |
| Ciliated cells | 228 | 77 | 1.64E-20 | -0.2477011 |
| Plasmacytoid dendritic cells | 66 | 0 | 9.37E-03 | Inf |
| All | 57500 | 4868 |  |  |

**Table S1. Percentage of different cells in MPLD and SPLD.**
